# Cardiac papillary fibroelastoma: a retrospect of four cases

**DOI:** 10.1186/1749-8090-8-65

**Published:** 2013-04-05

**Authors:** Mi Zhang, Xiaohong Liu, Zhigang Song, Liangjian Zou, Bo Xiang

**Affiliations:** 1 Department of Cardiothoracic Surgery, Changhai Hospital, Second Military Medical University, No.168, Changhai Road, Yangpu District, Shanghai 200433, China; 2 Institute of Thoracic and Cardiac Surgery, Changhai Hospital, Second Military Medical University, No.168, Changhai Road, Yangpu District, Shanghai 20043, China

**Keywords:** Fibroelastoma, Echocardiography, Cardiovascular event

## Abstract

**Objective:**

We have reviewed the medical histories of 4 patients who underwent operations between November 2004 and February 2011 at Changhai Hospital for cardiac papillary fibroelastoma.

**Methods:**

Diagnosis was demonstrably suggested by echocardiography. Tumor locations were mitral valve (1), left atrium (1), and aortic valve (2). Indications for operation were previous cerebrovascular accident for the mitral tumor, incidental apopsychia and giant mobile mass for the left atrium, ingravescent chest tightness and palpitations for the first aortic tumor, and severe regurgitation of aortic valve for the second aortic tumor. The study was approved by the Changhai Hospital Ethics Committee, and the consent from the patients or their immediate family was obtained.

**Results:**

Surgical excision with necessary valve replacement operations was performed in all cases. All patients had uneventful postoperative recoveries. No evidence of regurgitation or recurrence was seen on echocardiography at follow-up.

**Conclusions:**

Despite their histologically benign aspect, cardiac papillary fibroelastomas should be removed because of potential embolic complications.

## Background

Cardiac papillary fibroelastoma (CPFE) is rarely in literature as a primary cardiac neoplasm of unknown pathogen [1]. The incidence of primary cardiac tumors is reported to be less than 0.3% in autopsy series, and approximately 50% of them are cardiac myxomas [2,3]. CPFE, taking the third place of the primary cardiac neoplasm, generally involves and damages the cardiac valves. As previously documented, though many papillary fibroelastomas may not cause symptoms, early diagnosis of CPFE is of prior importance to prevent patients from fatal complications [4].

We report 4 cases in this paper to demonstrate the complications, pathological characteristics of CPFE and the intraoperative findings.

## Case presentation

**Figure 1:**
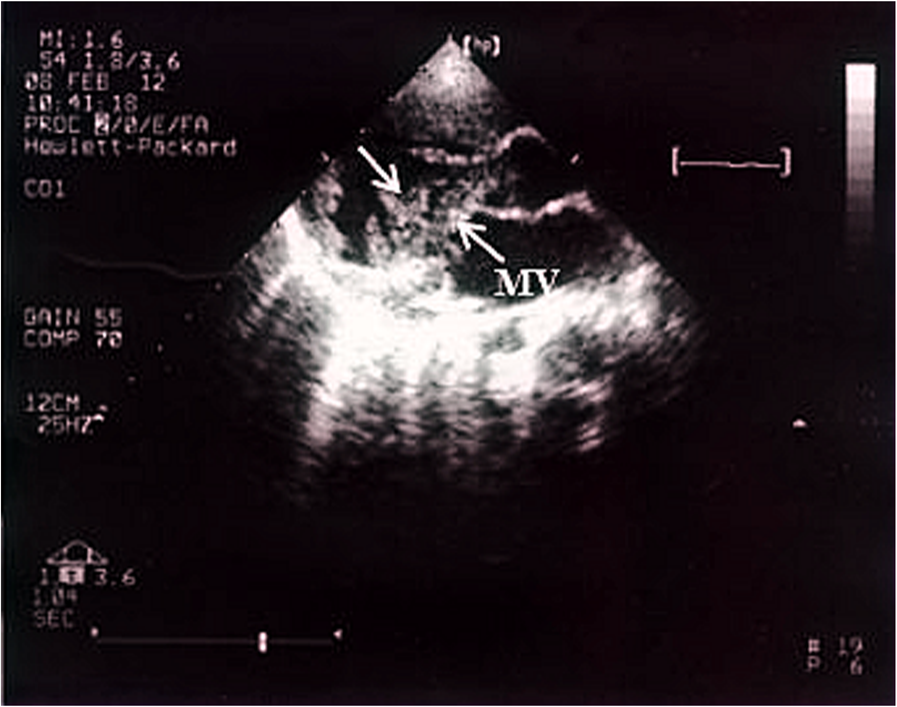
**Preoperative echocardiography image of the CPFE adherent to the mitral valve.** MV: mitral valve.

**Figure 2:**
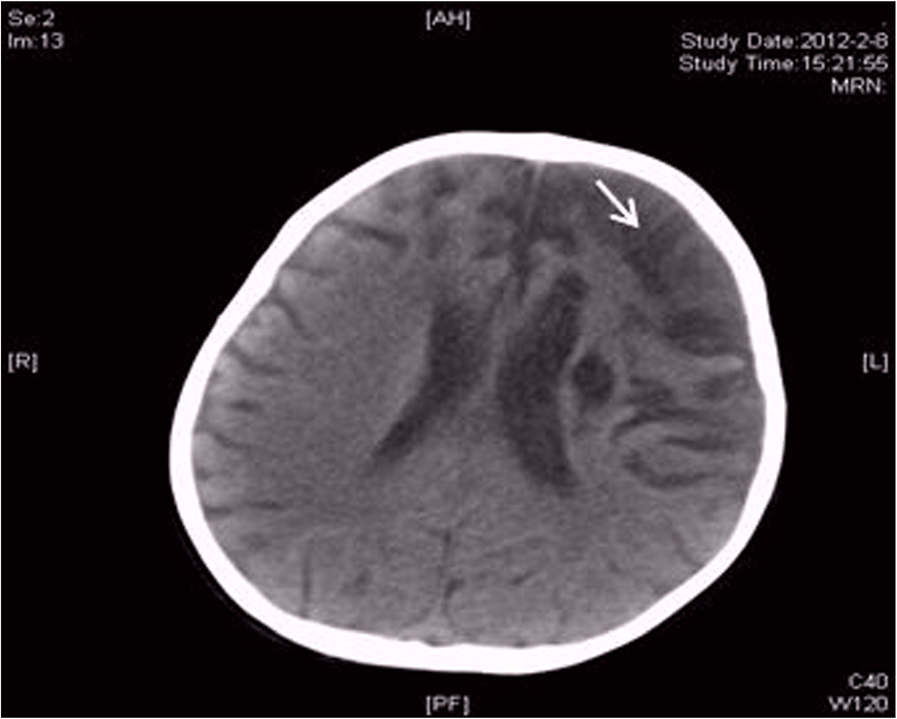
CT revealed low density imaging in the left parietal-temporal lobe, left basal ganglia and right frontal parietal-temporal lobe.

Patient 1: A 23-month-old baby girl was admitted to our hospital for the treatment of an ingravescent right side hemiplegia with prolonged low-grade fever. The patient was diagnosed 3 months ago as 1. cerebral infarction, acute right hemiplegia; 2. infective endocarditis; 3. respiratory tract infection. At cardiac examination, the patient was in sinus rhythm while presenting a II/ 6 systolic murmur in heart apex. Physical examination showed muscle strength decrease, hypermyotonia and positive Babinski sign in her right extremities. The right knee and tendo calcaneus reflexes hyperfunctioned. Transthoracic echocardiography revealed multiple mobile masses on the mitral valve (Figure 1), left ventricle and left ventricular outflow tract. The cranial computerized tomography revealed cerebromalacia in the left parietal lobes, left temporal lobes, left basal ganglia, right frontal parietal and right temporal lobes (Figure 2). Electrocardiogram showed an old myocardial infarction in inferior heart wall. Hemoculture was negative. After an effective anti-infective therapy, an operation was performed on February 13th, 2011. Surgical findings include multiple mobile masses attaching to mitral valve and left ventricle and tubular sclerosis in the distal part of left anterior descending artery. Neither patent ductus arterious nor other congenital cardiac anomalies existed. The tumor and the mitral valve was excised and replaced with an artificial heart valve. The following histopathology examination confirmed the diagnosis of a CPFE by gross image, hematoxylin-eosin and Victoria blue-van Gieson staining.

**Figure 3:**
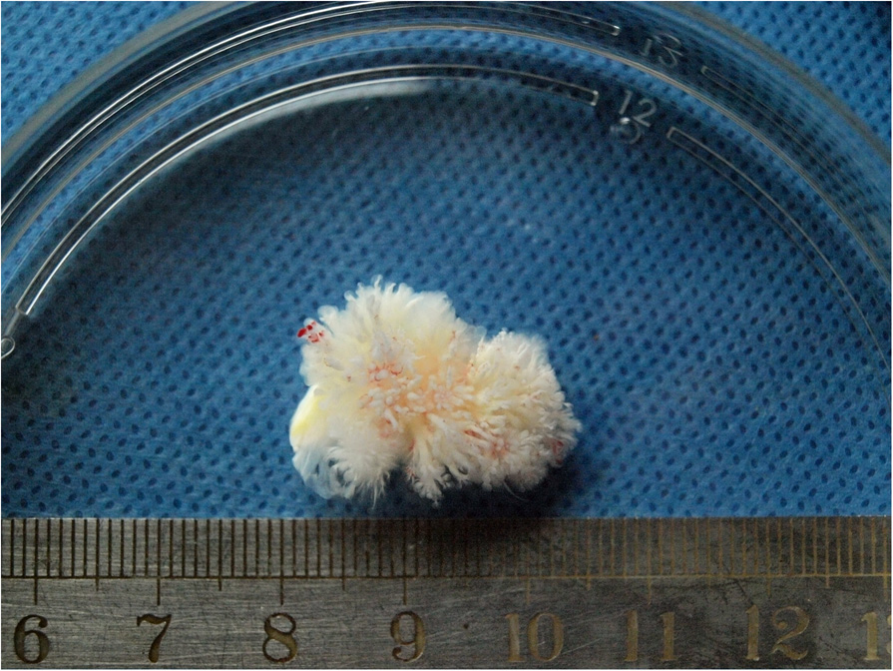
Gross image of a CPEF.

Patient 2: A 70-year-old woman was referred in April 2nd, 2006 for the treatment of an ingravescent dyspnea, palpitation and incidental apopsychia, especially arising after exercises. These symptoms cannot be controlled with an oral medication. The physical examination did not revealed any noticeable abnormalities. Transthoracic echocardiography revealed a comparatively hyperechoic loosen mass (24 × 25 mm)in the left atrium attached to the endocardium by a pedicle. Results of biologic, electrocardiographic, chest radiographic, and abdominal duplex ultrasonographic studies were all within normal limits. An operation was performed on April 7, 2006. Surgical findings consisted of a gelatinous mass, which is attached to the left atrium endocardium near the left auricle and a regurgitation of tricuspid valve. Papillary fronds of the tumot are narrow elongated, and branching (Figure 3). The patient underwent a tumorectomy along with an aortic valve replacement and a tricuspid valvuloplasty. The patient’s postoperative course was uneventful. The postoperative pathological studies confirmed the diagnosis of CPFE.

Patient 3: A 60-year-old woman was referred in June 23rd, 2011 for the gradually developing chest tightness and palpitations. The patient had an uneventful previous medical history except for an atrial fibrillation. She had been admitted to her local hospital because of previously described symptoms while she was resting in 2006 and 2007. Results of preoperative studies were all within normal limits. The electrocardiographic showed an atrial fibrillation. Meanwhile, the transthoracic echocardiography revealed a mobile mass (approximate 6 × 6 mm) attached to the left coronary valve accompanying atrioventricular valves regurgitation and pericardial effusion. The patient underwent a surgical resection in July 1st, 2011. The intraoperative finding consisted of a CPFE (confirmed by histologic examination) adhered to the free edge of the noncoronary aortic cusp. The tumor and the aortic valve were removed. An aortic valve replacement was then performed.

Patient 4: A 34-year-old woman was referred in November 11th, 2004 for a cardiac murmur newly revealed in a physical examination which is undertaken for an acute upper respiratory tract infection. At admission, results of clinical examination included a systolic murmur in aortic valve auscultation II along with a diastolic murmur in aortic valve auscultation. Transthoracic echocardiography revealed 1. supracristal ventricular septal defect (approximate 7 mm in diameter ); 2. aortic valve malformation with severe regurgitation. The aortic valve replacements combined with septal defect morioplasty was accomplished on November 26th, 2004. Intraoperative foundings accorded with the echocardiography diagnosis. The postoperative tissue biopsy showed a neoplasm (around 2 mm in its greatest dimension), that is of a sea anemone-like appearance with multiple papillary fronds attached to the endocardium by a short pedicle. The tumor with a single layer of endocardial cells covering the papillary surface merged imperceptibly into the subendothelium of the noncoronary cusp. Microscopically, the hyperplastic compact collagen embedded in loose connective tissue composed the core of this tumor. The elastic fibers were irregular and fractured. In view of the pathological foundings, a CPFE was diagnosed.

## Conclusions

After Cheitlin et al. [5] defined this tumor as “papillary fibroelastoma” for the first time, CPFE was sporadically reported with an incidence between 0.002% and 0.33% at autopsy ranging [6]. Male seems to be more vulnerable [1]. However, it is contradicted that all of our 4 patients are female. The tumor always occurs sporadically in all age groups, but it clearly predominates in adults. The average age of diagnosis is around 60 [7]. It is extremely rare in children. Therefore, the diagnosis of a CPFE in infants and children is of vital importance due to a higher risk for embolization in this age group [8]. Notably, the youngest patient here is only around 2 years old. Considering her uneventful past medical history, it is quite evident to make a diagnosis of congenital CPFE. Unfortunately, in this patient a cerebral embolization occurred before the excision was performed.

Grossly, CPFE has a distinctive flower-like appearance with multiple papillary fronds attached to the endocardium by a short pedicle [9]. Papillary fronds are narrow elongated, and branching. They merge imperceptibly into the substance of the valve, and are easily visible when immersed in water or saline solution to show a distinctive sea anemone-like appearance, thus providing easy morphological verification of the tumor being a CPFE (Figure 3).

**Figure 4:**
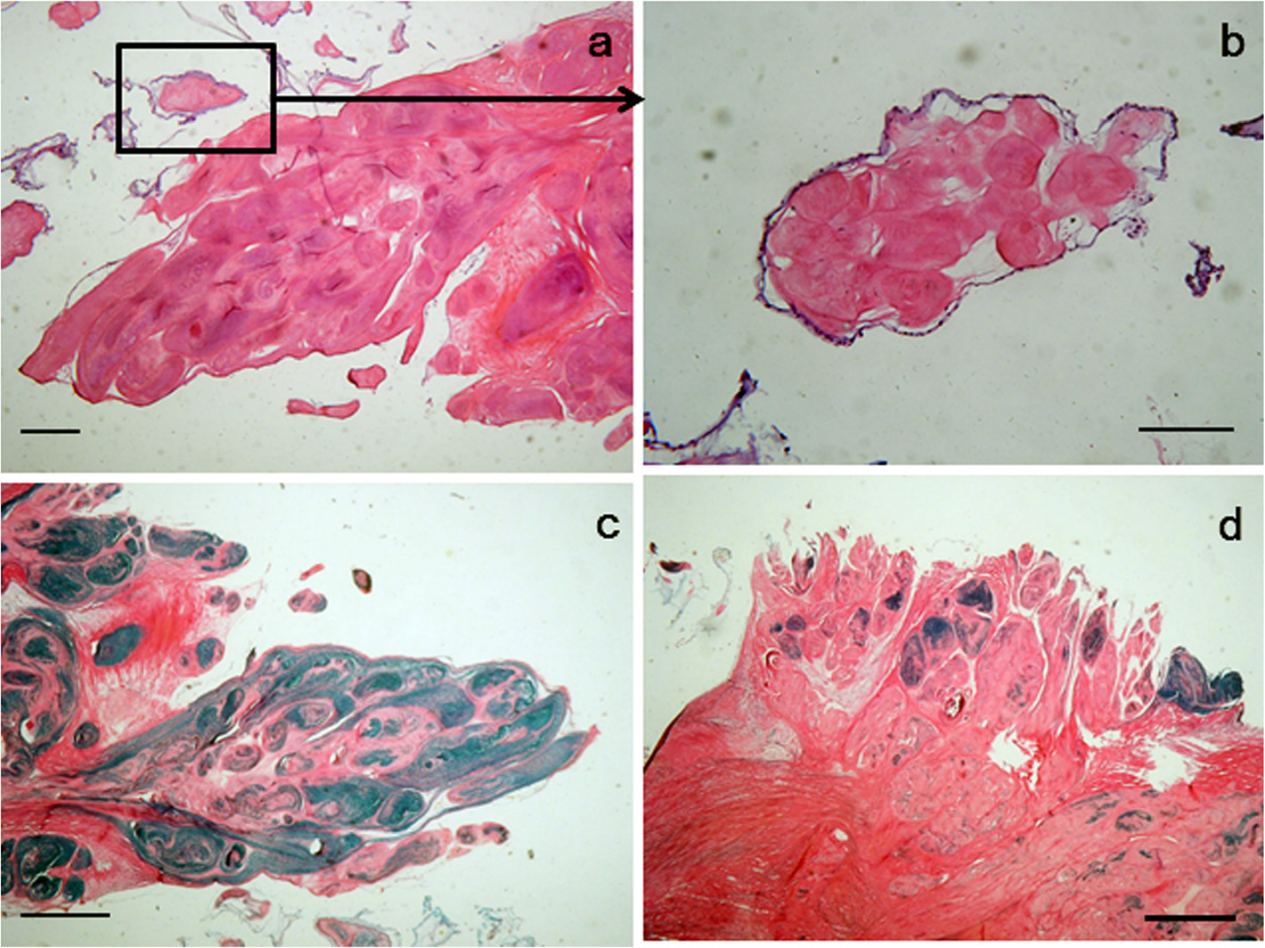
**Histologic section of an excised CPFE.** (**a**) CPFE has a distinctive flower-like appearance with multiple papillary fronds. (**b**) The papillaryfronds are covered by endothelial cells. (**c**, **d**) Elastic fiber varies in tumor substance. (bar = 100 μm).

CPFE is a microscopically avascular papillomass (Figure 4a) with a single layer of endocardial cells covering the papillary surface (Figure 4b). Matrix consists of proteoglycans and elastic fibers, but their distribution could be variable. Elastic Victoria-blue and van Gieson stain is especially useful for delineating these components (Figure 4c, d).

Most CPFE are asymptomatic and found incidentally at the time of routine physical examination, echocaidiography, cardiac catheterization, cardiac surgery or autopsy. CPFE usually develop on cardiac valves, but many arise anywhere else in the heart. It present with a great variety of symptoms, especially cardiovascular in nature [7,10-13]. The clinical presentation is determined by many factors, including tumor location, size, growth rate, and tendency for embolization. The most common fetal complications are caused by embolism to cerebral, systemic or coronary arterial circulation, followed by heart failure and sudden death [14-17]. In our review, emboli had led to cerebrovascular and cardiovascular accident in Patient 1, who presented a group of symptoms resembling infective endocarditis.

Sun et al. [7] recommend the following management of patients with CPFE: 1. In asymptomatic patients with right-sided CPFE, a conservative strategy is acceptable except for the large mobile tumors; 2. The presence with a sizeable right-to-left shunt is a risk factor for right-sided CPFE; 3. Asymptomatic patients with small, left-sided, nonmobile CPFE are usually observed; 4. CPFE ≥ 1 cm, especially, if mobile, should be considered for excision; 5. Patients with other cardiovascular disease, low risk of surgery and a high risk for embolization is recommended for an operation. But, others argued, prophylactic tumor excision with valve replacement when necessary, even in asymptomatic patients, is considered by many to be the treatment of choice [13,18].

In our experience, these tumors, even in asymptomatic patients with small-sized, should be surgically managed. Because even tiny CPFE can lead to stroke and other irreversible complications, like in Patient 1. The severe consequence indicates that in young patients, wherever dose a CPFE locate, the surgical resection should be the primary therapy strategy after careful evaluation to eliminate the life-long threaten of embolism. Mechanical damage to a heart valve or adhesion of the tumor to valve leaflets may call for valve repairmen or replacement.

## Consent

Written informed consent was obtained from the patients or their legal guardians for publication of this case report and any accompanying images. A copy of the written consent is available for review by the Editor-in-Chief of this journal.

## Competing interests

All authors declare that they have no competing interests.

## Authors’ contributions

All authors have contributed in case management, manuscript preparation and image acquisition. All authors read and approved the final manuscript.
